# Chromosome-level genome of *Mosla chinensis* from alpine ecotype provides insights into terpenoid biosynthesis and germplasm exploration

**DOI:** 10.3389/fpls.2026.1819008

**Published:** 2026-06-16

**Authors:** Zong-Xia Yu, Hai-Feng Wang, Ruo Lv, Shi-hao Liu, Wan-Bo Zhang, Xi Liu, Yong-Hua Zhang, Pan Li, Xin-Jie Jin

**Affiliations:** 1 Jiangxi Key Laboratory for Sustainable Utilization of Chinese Materia Medica Resources, Lushan Botanical Garden, Jiangxi Province and Chinese Academy of Science, Jiujiang, China; 2 Shanghai Key Laboratory of Plant Functional Genomics and Resources, Shanghai Chenshan Botanical Garden, Shanghai, China; 3 College of Life and Environmental Science, Wenzhou University, Wenzhou, China; 4 Wuyanling National Nature Reserve Management Center of Zhejiang, Wenzhou, Zhejiang, China; 5 Institute for Eco-environmental Research of Sanyang Wetland, Wenzhou University, Wenzhou, China; 6 Key Laboratory of Biodiversity and Environment on the Qinghai‐Tibetan Plateau, Ministry of Education, School of Ecology and Environment, Xizang University, Lhasa, China

**Keywords:** alpine ecotype, chromosome-scale genome assembly, evolutionary analyses, *Mosla chinensis*, terpenoid biosynthesis

## Abstract

*Mosla chinensis* is a high-value medicinal plant with scarce genomic resources for precision breeding. Here, we present the first chromosome-scale genome of an alpine ecotype (Baiyunjian Reserve, 1,611 m above sea level) assembled via SMRT sequencing and Hi-C technologies. 93.46% of sequences were anchored to 9 pseudochromosomes with 99.99% accuracy, resulting in the assembly of the 444.26 Mb genome (contig N50 = 41.38 Mb; 98.7% BUSCO). High proportion of repetitive sequences (56.90%) and species-specific genome duplication revealed the species-specific adaptations of this ecotype. Multi-tissue metabolomics identified flowers and leaves as biosynthetic hotspots for terpenoids especially γ-terpinene, thymol and carvacrol. Integrated with transcriptomics pinpointed MchTPS4 as the plastid-localized γ-terpinene synthase, while phylogenetic mining identified cytochrome P450 MchCYPs (MCH03g18750) and dehydrogenases MchSDRs (MCH04g17930/14560) as potential downstream enzymes. Functional validation confirmed that MchTPS4 was a monoterpene synthase while MchTPS2, MchTPS7, MchTPS9–11 were sesquiterpene synthases. Furthermore, metabolic engineering boosted the yield of γ-terpinene 3.45-fold to 5.09 mg/L. Biosynthetic gene clusters (BGCs) analyses uncovered 61 clusters with 11 associated with terpenoid metabolism. MchTPS2 co-localized in cluster 6 with two MchCYPs (MCH01g19200; MCH01g19220). Syntenic analysis revealed that MchTPS2 evolved as a single-copy, whereas MCH01g19200 underwent tandem duplication from *M. chinensis* to *Salvia divinorum* and *S. hispanica*. Phylogenomic analysis resolved *M. chinensis* as the sister species of *Perilla frutescens* (100% BS), diverging ~12.7 Mya in the mid-Miocene. Comparative genomics revealed a species-specific whole-genome duplication event post-dating divergence from *Scutellaria baicalensis*, driving syntenic retention of specialized metabolic genes. This study decodes the genetic basis of terpenoid biosynthesis in an extremophyte, providing resources for molecular breeding of traditional Chinese medical plant “*Xiangru*” and insights into Lamiaceae metabolic evolution.

## 1 Introduction

*Mosla chinensis* Maxim is an annual herb widely distributed in southern China, Korea, and Vietnam (Jang et al., 2022; Jin et al., 2025; Wu, 1977). Its phylogenetic position has been clarified in recent taxonomic studies, confirming that it belongs to the genus *Mosla*, one of the seven genera in the tribe Elsholtzieae (Lamiaceae) (Jin et al., 2025; Li et al., 2017). Beyond its phylogenetic significance, *M. chinensis* has long been recognized for its medicinal properties, with its earliest historical record found in the *Ming Yi Bie Lu* from the Wei and Jin Dynasties (Li et al., 2014), and continues to be widely utilized in the therapy of cold, diarrhea, digestive disorders and edema in traditional Chinese medicine (Commission, 2020). And its variety, *M. chinensis* cv.*’Jiangxiangru’*, is ranked as one of the ten genuine medicinal materials (also known as Dao-di Herbs) in Jiangxi Province of China (Luo et al., 2021; Wang et al., 2021). In the *Pharmacopoeia of the People’s Republic of China*, “Xiangru” refers to *M. chinensis* and *M. chinensis ‘Jiangxiangru’* (Commission, 2020). In addition to pharmaceutical, *M. chinensis* is also widely applied in food, active packaging, and cosmetics industry because of its abundant essential oils (Gao et al., 2023). Terpenoids, especially iridoid monoterpenoids such as thymol and its isomer carvacrol, are the major components of the essential oils, which exhibit well-documented antioxidant, antibacterial, anti-inflammatory, insecticidal, and immune-enhancing activities (Feng et al., 2024; Lu et al., 2020b; Wang et al., 2024).

Terpenoid biosynthesis, including the synthesis of monoterpenes, follows evolutionarily conserved upstream pathways consisting of the mevalonic acid (MVA) and methylerythritol phosphate (MEP) pathways (Nagegowda and Gupta, 2020; Vranova et al., 2013). The biosynthesis of terpenoids is initiated with the synthesis of five-carbon isoprene building blocks, isopentenyl diphosphate (IPP) and dimethylallyl diphosphate (DMAPP), via the MVA and MEP pathways (Lipko et al., 2023; Vranova et al., 2013). Subsequently, the precursors of monoterpene (C10), sesquiterpene (C15) and diterpene (C20) are condensed from different number of IPP and DMAPP (Vranova et al., 2013). However, the downstream of terpenoid synthesis is diverse. Specific terpenoid synthases (TPS) catalyze the corresponding precursors to form different terpenoid skeletons, which can be further modified into various terpenoid derivatives by cytochrome P450 monooxygenase, glycosyltransferases and so on (Lu et al., 2023; Zhang et al., 2023).

Although there is a certain understanding of the upstream pathways of terpene biosynthesis, the functional genes in *M. chinensis* have not been fully studied, especially the key genes related to terpene biosynthesis. The downstream biosynthetic pathways of thymol and its isomer carvacrol have long been a mystery for over 40 years. According to previous reports, there are two possible pathways for their synthesis. One proposed pathway is that thymol is synthesized from geranyl diphosphate (GPP), the ubiquitous precursor of monoterpenes, with γ-terpinene and p-cymene as the intermediates. This is supported by the evidence that exogenous γ-[G-^3^H] terpinene could be converted into p-cymene and thymol, whereas p-[G-^3^H] cymene produced only thymol in the leaves of thyme (*Thymus vulgaris* L.) (Poulose and Croteau, 1978). The other proven pathway is that under the catalysis of TPS, GPP is cyclized into γ-terpinene, which is oxidized by cytochrome P450 monooxygenases of the CYP71D subfamily, and then dehydrogenated by a short-chain dehydrogenase/reductase (SDR) to thymol and carvacrol (Krause et al., 2021). Thus, the biosynthesis pathway of thymol and carvacrol has been elucidated in oregano (*Origanum vulgare* L.) and different *Thymus* species in the Lamiaceae (Crocoll et al., 2010; Tohidi et al., 2020). Lately, the biosynthesis pathway of thymol and carvacrol was also unveiled in *M. chinensis* via genome sequencing and assembly (Yu et al., 2025). However, there are still plenty of terpenoids that need to be explored in *M. chinensis*, from catalytic enzyme genes to regulatory networks, and this will enrich the gene pools for biosynthetic chassis construction as well as molecular breeding.

To effectively discover functional genes, systematic gene family classification and evolutionary analysis are crucial, as they not only help identify functionally similar members with diverse products but also provide strong support for functional predictions. A specific gene family usually has a common phylogenetic origin. Members of the same gene subfamily show sequence and function similarities yet product diversities (Aubourg et al., 2002). Therefore, phylogenetic analysis is an effective way to narrow down candidates through functional prediction. To explore terpenoid biosynthesis, both TPS and modification gene families such as CYP and SDR play critical roles. TPS family is classified into six subfamilies. Among them TPS-b and TPS-g subfamilies mainly catalyze the synthesis of monoterpenes (Zhou and Pichersky, 2020). In the CYP superfamily, the subfamilies CYP71, CYP76, CYP706, CYP736, and CYP750 of the CYP71 clan possess the function of oxidizing terpenoids (Hansen et al., 2021). However, the SDR superfamily shows low sequence identities but is characterized by a conserved “Rossman-fold” 3D structure. Among its members, those from the SDR110C, the SDR25C, SDR114C, SDR460A subfamilies involve in the synthesis of terpenoids (Moummou et al., 2012).

This research presents the chromosome-level genome assembly of the alpine ecotype of *M. chinensis*, which possesses unique metabolic and genetic characteristics for developing germplasm with high resistance to extreme environments, through high-fidelity sequencing (HiFi) and high-throughput chromatin conformation capture (Hi-C). Gene structure analysis, gene phylogenetic tree construction, transcriptome profiling and enzyme activity assay were conducted to identify different enzyme genes and verify the functions of TPS. Species phylogenetic tree construction, intragenomic and intergenomic collinear analyses, BGCs mining were performed to uncover the evolutionary history of the species and terpenoids. Since this alpine ecotype of *M. chinensis* is an exceptional variant that has adapted to high-altitude and harsh environmental conditions, our genome assembly, gene functional characterization, and comparative genomic analyses will facilitate molecular breeding aimed at enhancing the therapeutic component content and stress resistance of this species. Ultimately, this work will support the growing demand for *M. chinensis* in the traditional Chinese medicine (TCM) industry through genetic improvement and industrial applications.

## 2 Methods

### 2.1 Karyotype analysis and genome size evaluation of *M. chinensis*

*M. chinensis* plants were collected from the Baiyunjian Reserve (1,611 m elevation) in Taishun Town, Wenzhou City, Zhejiang Province, and cultured in the lab for one generation before sequencing. The seeds were placed on moist filter paper in a glass petri dish and cultured in a refrigerator (0-4 °C) for 3–5 days. Then the petri dish was taken out of the refrigerator and placed at room temperature (20-25 °C) for germination. Collected the root tips from the 1–2 cm length roots. Soaked in the 0.002 M 8-hydroxyquinoline solution at 25 °C for 3 hours under dark conditions, then rinsed with ultrapure water (UP water) for 5 times. Fixed the root tips with Carnoy’s fixative solution (ethanol:glacial acetic acid = 3:1) on ice for 2 hours, then rinsed with UP water for 5 times. Resolved the root tips in a mixture of 45% glacial acetic acid (v/v) and 1 mol/L HCl (volume ratio 1:1) in a 60 °C water bath for 5 min and rinsed with UP water for 5 times, followed by sinking in UP water for 2 hours. Stained with carbol fuchsin solution for 10–15 min and then prepared the slides. Observed under a 20× objective lens of a LEICA DM1000 optical microscope, and captured images under a 100× objective lens.

The genome size of *M. chinensis* was primarily estimated by flow cytometry (BD FACScalibur) using *Solanum lycopersicum* (900 Mb) and *Zea mays* B73 (2.3 Gb) as internal standards, with data analyzed by Modifit3.0 software. As a supplementary cross-validation, a 17-bp k-mer analysis was performed using Jellyfish (v.2.0) based on Illumina sequencing data (a 150 bp insert-size DNA library was constructed with Illumina DNA Prep Kit and sequenced on Illumina NovaSeq 6000 platform using 2×150 bp paired-end strategy), from which genome size was preliminarily inferred. The total number of k-mers (n_kmer) was 22,769,553,690 counted using SOAPdenovo software. The k-mer depth was estimated to be 48× by fitting a Poisson distribution. The initial estimated genome size was 474.37 Mb (Genome size = n_kmer/k-mer depth), and the revised genome size after correcting for erroneous k-mers was 465.74 Mb.

### 2.2 Genome sequencing, assembly and annotation

A SMRT library containing about 15–18 kb cut fragment was constructed and sequenced by PacBio sequel II platform for the long reads, and 23 G data was obtained. Illumina HiSeq platform was used for short read sequencing, and 30 G data was obtained. The genome was sequenced by Novogene Co., Ltd.

A total of 53.00 G of sequencing data (113.98× coverage) was used for *de novo* genome assembly by hifiasm software with the default parameters (Cheng et al., 2021). Hi-C libraries were constructed following the standard *in situ* Hi-C protocol. Briefly, chromatin was cross-linked, digested with DPNII, and the resulting DNA fragments were labeled with biotin, followed by proximity ligation. After reversing crosslinks, DNA was purified, sheared (350 bp), and biotinylated fragments were captured for library preparation. A total of 23.4 G data were used for the chromosomal-level scaffolding from ALLHiC (v0.9.8) following a five-step pipeline: (1) Pruning to remove allelic and homologous cross-links; (2) Partition to cluster contigs into chromosome groups based on interaction frequencies; (3) Rescue to reassign unclustered contigs; (4) Optimization using genetic algorithm for ordering and orientation; and (5) Building to generate chromosome-scale assembly. The resulting assembly was manually corrected using Juicebox (v1.11.08) based on the heatmap of the interaction intensity and relative position between contigs (Robinson et al., 2018). The contigs were finally linked into nine pseudochromosomes.

Homology alignment and *de novo* search were combined for the repeat annotation. RepeatMasker software was used to search against Repbase database to extract repeat regions (Tempel, 2012). A *de novo* repetitive elements database was constructed by RepeatModeler, which was supplied to RepeatMasker for DNA-level repeat identification (Price et al., 2005). Structural annotation incorporated ab initio prediction, homology-based prediction and RNAseq assisted prediction. Sequences of six relative species (*Scutellaria baicalensis*, *Salvia miltiorrhiza*, *Arabidopsis thaliana*, *Perilla frutescens*, *Solanum pennellii*, *Nicotiana tabacum* were aligned to the genome to predict gene structure using TblastN (v2.2.26; E-value ≤ 1e^−5^) and GeneWise (v2.4.1) softwares (Birney et al., 2004; Kent, 2002). Augustus (v3.5) and SNAP were used for ab initio prediction (Stanke et al., 2006). RNAseq reads were aligned to genome fasta using HISAT2 (v2.2.1). The alignment results were put into Stringtie (v2.2.1) for genome-based transcript assembly (Haas et al., 2003). The gene predictions from the three methods were integrated using EvidenceModeler (EVM, v1.1.1) to generate a non-redundant reference gene set (Haas et al., 2008), which was further corrected with PASA (Program to Assemble Spliced Alignments).

Functional annotation of the predicted protein-coding genes was carried out by performing Blastp (E-value ≤ 1e^−5^) against the Swiss-Prot database. The motifs and domains were annotated using InterProScan70 (v5.39) by searching against ProDom, PRINTS, Pfam, SMRT, PANTHER and PROSITE databases (Zdobnov and Apweiler, 2001). The Gene Ontology (GO) IDs for each gene were assigned according to the corresponding InterPro entry (Ashburner et al., 2000). Pathway annotation was performed using KOBAS (v3.0) against the KEGG database (Kanehisa and Goto, 2000).

### 2.3 Analyses of gene structure, domain and conserved motif compositions

Initially, based on the GFF3 file format, we extracted and analyzed the exon-intron structure of the target gene. For the analysis of conserved domains, we utilized the Batch Conserved Domain Search tool available on the NCBI website (https://www.ncbi.nlm.nih.gov/Structure/bwrpsb/bwrpsb.cgi) (Lu et al., 2020a). The analysis was conducted using default parameters, including an E-value threshold of 0.01 and low-complexity region filtering, to ensure the reliability of the results. This tool, based on the CDD database, employs the RPS-BLAST algorithm for the identification and annotation of conserved domains. We employed the MEME (Multiple Em for Motif Elicitation) online analysis tool (https://meme-suite.org/meme/tools/meme) for protein motif analysis. The parameters were set as follows: the maximum number of motifs was set to 10, the motif distribution pattern was set to “Any Number of Repetitions”, and other parameters were kept at their default settings. The MEME algorithm, based on the Expectation Maximization (EM) principle, effectively identifies conserved functional motifs in protein sequences, providing crucial insights for protein function prediction. We employed TBtools software (v2.154) for comprehensive data integration and visualization of the analytical results (Chen et al., 2020).

### 2.4 Chromosome distribution, collinearity analysis and BGCs discovery

The chromosomal localization of genes was obtained from the General Feature Format (GFF3) file and visualized using the “Gene Location Visualize from GTF/GFF” function in TBtools software. To analyze the collinearity among the nine chromosomes, we employed the “One Step MCScanX - Super Fast” module in TBtools (Chen et al., 2023). Based on the physical location information of the genes derived from the analysis, we utilized the “Advance Circos” function in TBtools to display the results. The chromosome-scale assembly of *M. chinensis* (nine chromosomes) was visualized in a circular genome plot. Collinearity analysis was conducted by comparing *M. chinensis* with itself or with *S. baicalensis* (Accession No. GWHAOTO00000000) and *S. officinalis* (Accession No. GWHBJVP00000000). The genome information were analyzed by “One Step MCScanX - Super Fast” module and displayed by “Multiple Synteny Plot” module in TBtools. The genome data were uploaded to plantiSMASH (https://plantismash.bioinformatics.nl/) in order to discover the BGCs in *M. chinensis*. The default parameters with additional analysis of comparisons to plantiSMASH-predicted gene clusters were performed.

### 2.5 Transcriptome sequencing and analysis

The different tissues of *M. chinensis*, including the full-bloom flowers (OF), flower buds (CL), leaves (IL), stems (S) and roots (R), were sampled in triplicate for transcriptome sequencing. A total of 2 μg mRNA per sample was purified using Oligo dT magnetic beads. Quality control was verified by the Agilent Technologies 2100 bioanalyzer (Sunnyvale, CA, USA). The cDNA library was constructed, sequenced (Illumina platform) by Novogene Co., Ltd.

The brief analysis processes were as follows: the raw data were first processed using FastQC (Davis et al., 2021). Then, the clean reads were mapped to the *M. chinensis* genome with HISAT2 (Kim et al., 2019). The novel genes were predicted by StringTie (Pertea et al., 2016) and annotated by comparing to the public database such as Pfam, SUPERFAMILY, GO (Gene Ontology) and KEGG (Kyoto Encyclopedia of Genes and Genomes). The gene expression levels were normalized using FPKM (Fragments Per Kilobase of transcript per Million mapped fragments) (Li and Dewey, 2011). The gene expression levels of other tissues were compared to those of stems to identify DEGs (Differentially Expressed Genes, |log2(FoldChange)| >= 1 & padj<= 0.05). GO and KEGG were analyzed by clusterProfiler (Yu et al., 2025).

### 2.6 *MchTPS*, *MchCYP and MchSDR* genes identification and screen

To identify the *MchTPS*, *MchCYP and MchSDR* genes in *M. chinensis*, genes previously reported to be involved in thymol and carvacrol biosynthesis including *TvTPS2*, *OvTPS2*, *TcTPS2*, *CYP71D179* and *TvSDR1* from *T. vulgaris*, *T. caespititius*, and *O. vulgare* were used as queries to search against the protein database of *M. chinensis* via BLAST (Krause et al., 2021; Lima et al., 2013; Mount, 2007). Meanwhile, the Pfam accession numbers (PF01397 and PF03936 for TPS family; PF00067 for CYP family; PF00106, PF01370 and PF01073 for SDR family) were used as queries to search against the *M. chinensis* protein database using Advanced HMMER Search module in TBtools (Chen et al., 2023). Data retrieved from both BLAST and HMMER analyses were combined to form the initial candidate pools of *MchTPS*, *MchCYP and MchSDR* genes for subsequent screening. The improperly annotated and incomplete genes were further excluded via Simple MEME Wrapper module in TBtools and Web CD-Search Tool in NCBI (Chen et al., 2023; Wang et al., 2023).

To screen for potential candidate genes involved in terpenoids biosynthesis in *M. chinensis*, phylogenetic trees were first constructed using the One Step Build a ML Tree module in TBtools (Chen et al., 2020) to identify genes showing a close relationship with the functionally verified *TvTPS2*, *OvTPS2*, *TcTPS2*, *CYP71D179* and *TvSDR1*. The trees were optimized using the iTOL online software (Letunic and Bork, 2024). Subsequently, transcriptomes from different tissues of *M. chinensis* were analyzed. DEGs results were used to identify candidate genes exhibiting expression patterns consistent with the accumulation of terpenoids metabolites. Additionally, KEGG pathway enrichment analysis was conducted to discover candidate genes involved in terpenoid biosynthesis. The subcellular localizations of candidate genes was also considered. For instance, *TPS* genes that catalyze monoterpenoid synthesis are usually localized in plastids, while those that catalyze sesquiterpenoid synthesis are localized in the cytoplasm.

### 2.7 Single-copy nuclear gene discovery and phylogeny reconstruction

Single-copy orthologous nuclear genes (SCGs) were identified using OrthoFinder (v3.0.1b1) with default parameters (Emms and Kelly, 2019). Multiple sequence alignments of the SCGs were generated using MAFFT (v7.526), followed by trimming with trimAl (v1.5.rev0) under the default setting to remove poorly aligned regions (Rozewicki et al., 2019). The trimmed alignments were then concatenated into a supermatrix for maximum-likelihood phylogenetic reconstruction. Phylogenetic inference was performed on this supermatrix using IQ-TREE 2 (v2.4.0) under maximum likelihood, with automated model selection (ModelFinder Plus) and 1000 standard bootstrap replicates (Minh et al., 2020). Divergence times were estimated using the MCMCTREE program in PAML (v4.10.7) with fossil calibrations (Yang, 1997, Yang, 2007). Secondary calibration points for key nodes were obtained from the TimeTree (http://www.timetree.org), which integrates divergence time estimates from multiple studies calibrated with fossil evidence (Kumar et al., 2022). Given the lack of directly applicable fossil constraints for the studied clades, these secondary calibrations were adopted. For each node, divergence time ranges were used to define soft bounds in MCMCTREE, with all supporting references listed in (**Supplementary Table 1**).

### 2.8 Functional analysis of *MchTPSs*

In *E. coli*, *MchTPSs* were cloned into *pGEX-4T-1* and co-transformed with or without *pIRS* (over-expressing *DXS*, *DXR* and *IDI* genes to strengthen the MEP pathway of terpene synthesis) into *E. coli* BL21 (DE3). The positive clone was cultured in 600 μl of LB medium with optimal antibiotics with shaking (37 °C, 200 rpm) overnight. The culture was inoculated into 50 ml of TB medium containing antibiotics and cultured with shaking (37 °C, 200 rpm) until the OD_600_ reaches 0.6. Chilled on ice for 30 min and added 0.5 mM IPTG, 10 mM pyruvic acid, 1 mM MgCl2, 0.4% (v/v) glycerol and 20% (v/v) dodecane into the culture, then incubated with shaking (25 °C, 200 rpm) for 5 days. Added 10 ml of ethyl acetate into the culture, sonicated for 10 min, and shaken 30 min to extract the products. Centrifuge at 5000 rpm at 4 °C for 10 min to collect the supernatant, and diluted the supernatant with proper amount of hexane before GC-MS detection.

For functional elucidation *MchTPSs* in *Nicotiana benthamiana*, *MchTPSs* were constructed into *pEAT-HT* and ultimately transformed into *agrobacterium tumefaciens* GV3101. The positive clone was inoculated into 1 mL of LB medium with optimal antibiotics and cultured overnight with shaking (28 °C, 200 rpm). The overnight agrobacterial culture was inoculated into fresh LB medium with antibiotics at a ratio of 1:1000, and incubated until OD_600_ ≈ 0.8 with shaking (28 °C, 200 rpm). Collected the cells by centrifugation at 5000×g for 10 min at 4 °C and resuspend the sediment with MES buffer (10 mM MES, pH 5.6; 10 mM MgCl_2_; 100 μM acetosyringone), and repeated the steps twice. Finally, adjust the agrobacterial solution to OD_600_ ≈ 1.0. Mixed *MchTPS* strains with *tHMGR* over-expressing stain at a ratio of 1:1 (v/v) and incubated in the dark at 28 °C for 3 hours. Infiltrated the solution into the abaxial surface of fully expanded leaves of 4–5 week-old *N. benthamiana* with 1 mL needleless syringe. Placed the plants in dark conditions at 28 °C for 16–24 hours, then transferred to a light incubator (25 °C, 16 h light/8 h dark) for further 5 days. Collected the infiltrated leaves, weighed, freezed and crushed into powder, then added extraction buffer (V_hexane: V_ethyl acetate = 85:15) at a ratio of 0.1 g/1ml (with internal standard nonyl acetate at 10 ng/μl), and sonicated for 20 min. Centrifuged at 15000 × g for 5 min at 4 °C, the supernatant was detected by GC-MS for products.

### 2.9 Volatiles detection by GC-MS

The samples were injected GC-MS instrument in split mode (2 μL, split ratio 5:1) at an inlet temperature of 220 ˚C. The volatiles were separated by the column of Zebron ZB-5HT-INFERNO (30 m x 250 μm x 0.1 μm) in the carrier gas of helium with a flow rate of 1.2 mL/min. The program for detecting the metabolites extracted from *E.coli* was as follows: Hold at 50 °C for 2 min, increased to 100 °C at a rate of 10 °C/min and hold for 10 min; enhanced to 120 °C at 1 °C/min; increased to 220 °C at 20 °C/min and hold for 2 min. The program for detecting the metabolites extracted from tobacco was: Hold at 50 °C for 2 min, increased to 110 °C at a rate of 5 °C/min and hold for 2 min; enhanced to 120 °C at 1 °C/min and held for 2 min; enhanced to 170 °C at 5 °C/min and held for 2 min; increased to 220 °C at 20 °C/min and hold for 2 min. Nonyl acetate (10 ng/ul, Aldrich) was used as the internal standard, the contents of volatiles were calculated by comparing to the internal standard.

## 3 Result

### 3.1 Genome assembly and annotation of *M. chinensis*

**Figure 1:**
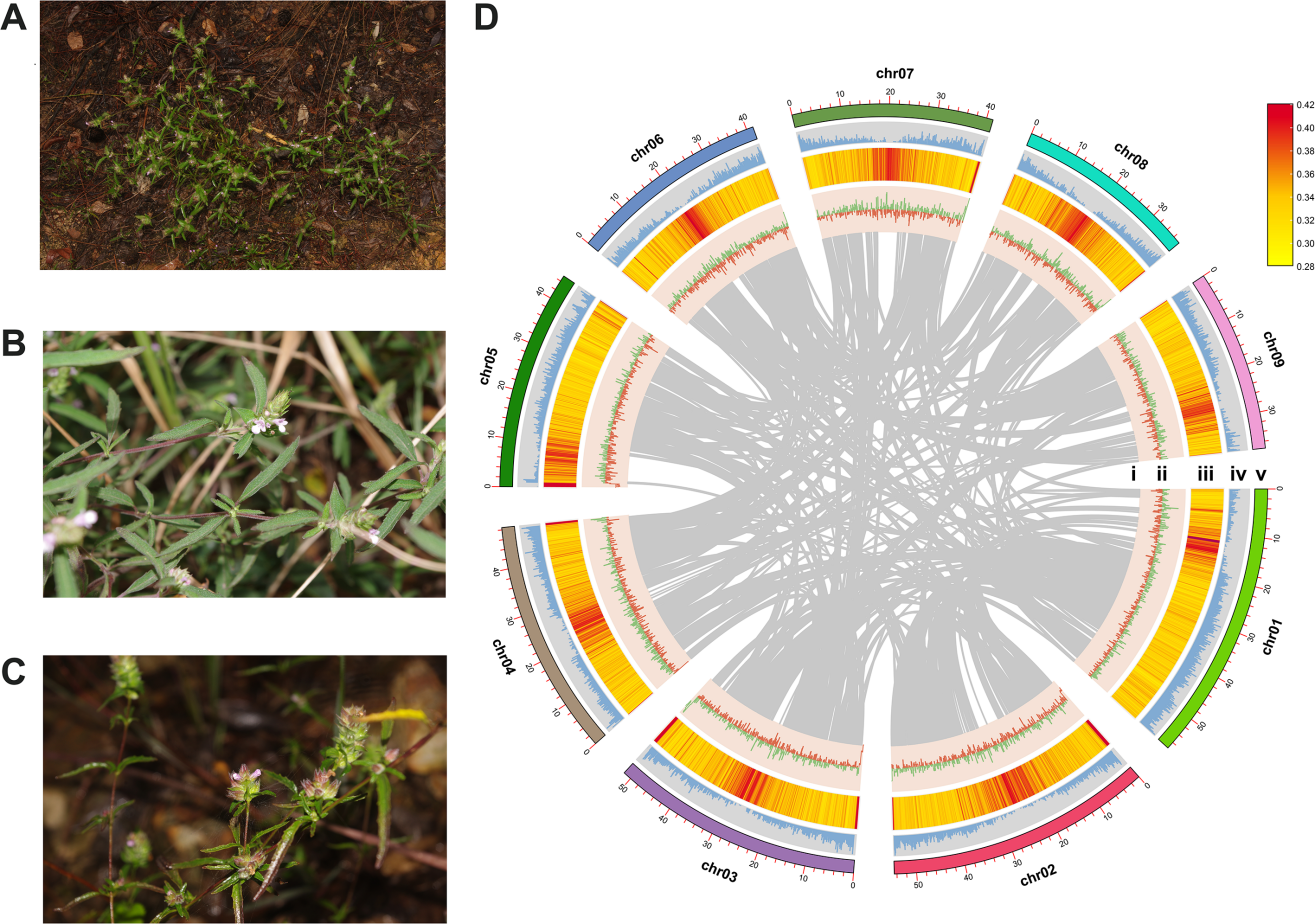
Habitat, morphology, and the chromosome-level genome of *M. chinensis*. **(A–C)** The habitat, leaves, and inflorescence of *M. chinensis* sequentially. **(D)** Overview of the chromosome-level assembly of *M. chinensis*. (i) Syntenic relationships among different chromosomes of *M. chinensis*. (ii) The GC skew along the genome. (iii) The GC content along the genome. (iv) The gene count along the genome. (v) The nine assembled *M. chinensis* chromosomes.

The size of the *M. chinensis* genome was predicted to be 481.90 Mb using flow cytometry (**Supplementary Figure 1**). A genome survey of *M. chinensis* using based on 30.96 Gb Illumina short reads estimated that the genome size could be 465.74 Mb with 0.20% heterozygosity and 57.59% repeat sequences. The genome size was 444.26 Mb with the contig N50 of 41.38 Mb and the scaffold N50 of 45.20 Mb. A total of 93.46% of the contig sequences (~415.18 Mb) were anchored to the nine pseudochromosomes with the length from 37 Mb to 57 Mb (**Figure 1**). The completeness of the *M. chinensis* genome was 98.7% evaluated by BUSCO (Benchmarking Universal Single-Copy Orthologs) (**Table 1**). The accuracy of the assembly was estimated by mapping the short reads to the assembled genome using the BWA software. The mapping rate was 99.55% and the coverage was 99.91%. The quality value (QV) of the genome was 45.23 assessed with Merqury mode, indicating the sequence accuracy was over 99.99%. Those parameters highlighted the high quality of *M. chinensis* genome.

**Table 1:** Statistics of *M. chinensis* genome assembly and annotation.

Feature	Statistic
Assembled genome size (Mb)	444.26
GC content (%)	34.97
Contig number	686
Contig N50 (bp)	41, 377, 152
Scaffold number	684
Scaffold N50 (bp)	45, 202, 222
Minimum len (bp) of contigs	14, 685
Maximum len (bp) of contigs	57, 481, 993
Mean len (bp) of contigs	647, 605
Number of annotated genes	25, 963
Repeats in genome (%)	56.9
Average BUSCO (complete) (%)	94.7

The annotations of the assembled genome mainly included repetitive sequences, genes, and non-coding RNAs (ncRNA). The genome was composed of 56.90% repetitive sequences and 0.82% ncRNA. A total of 26, 323 genes were predicted in *M. chinensis* genome using *de novo*, homology and RNAseq based analysis. And among them 25, 963 genes (98.63%) were functional annotated by searching the NR, Swissport, InterPro, Pfam, Gene Ontology (GO) and KEGG databases. The completeness of the assembled and annotated gene sets was 94.7% evaluated by BUSCO.

### 3.2 Metabolite profiling and transcriptome analysis of *M. chinensis* in different tissues

**Figure 2:**
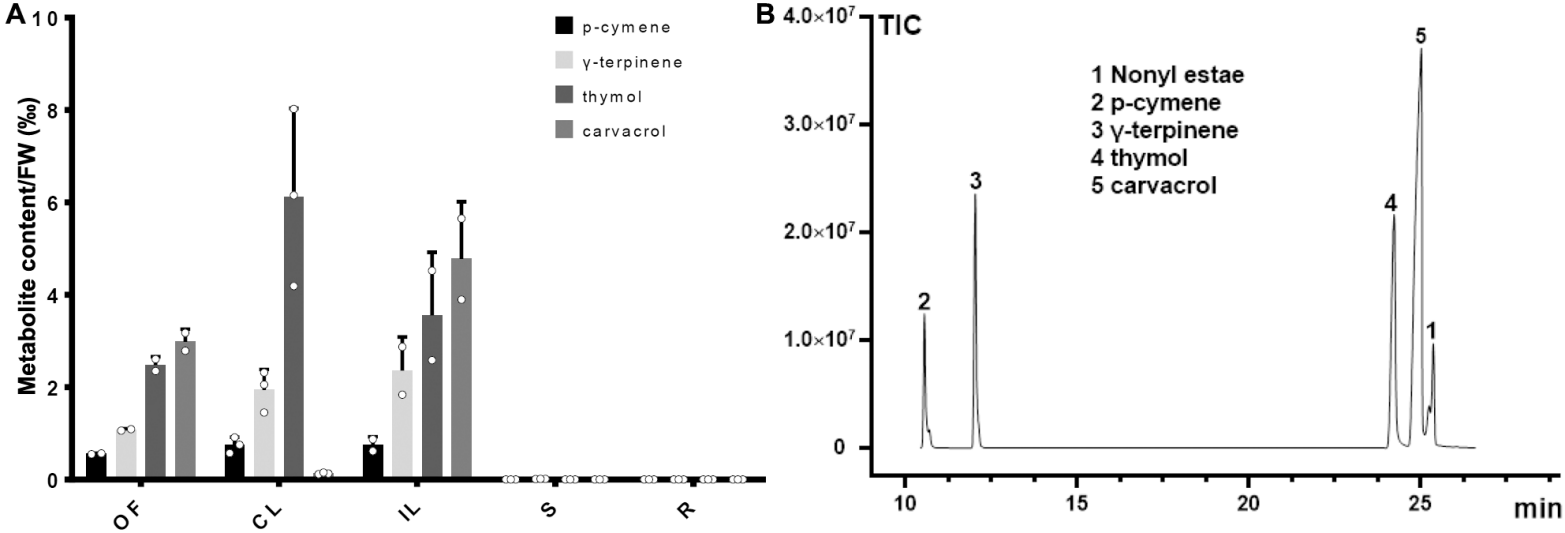
Volatiles of *M. chinensis*. **(A)** Contents of volatiles in different tissues. OF, full-bloom flowers; CL, flower buds; IL, leaves; S, stems; R, roots. **(B)** GC-MS trace of metabolites in the leaves. Nonyl acetate was used as the internal standard. Error bars represent the standard error (n=3).

To efficiently screen the candidate enzyme genes involved in terpenoids synthetic pathways, we performed GC-MS to explore their metabolite profiles in full-bloom flowers (OF), flower buds (CL), leaves (IL), stems (S) and roots (R) (**Figure 2**). The contents of major component of its essential oil, thymol and carvacrol, were significantly higher in the flowers and leaves than in the stems and roots. The contents of their precursors (γ-terpinene) and deduced intermediate (p-cymene) show similar patterns to thymol and carvacrol. Other terpenoids such as β-myrene, α-thujylalcohol, caryophyllene were also more abundant in flowers and leaves (**Supplementary Figure 2**).

Those aforementioned tissues were further analyzed by RNAseq to assist in the enzyme genes mining of terpenoids biosynthesis pathway. A total of 103.07 Gb raw data were obtained for those fifteen samples, and on average ~6.69 Gb clean data was generated for each replicate. The Q30 value was at least 91.62% indicating a good quality of the transcriptomes. A range from 82.98% to 94.21% of clean reads could mapped to the genome of *M. chinensis* (**Supplementary Table 2**).

**Figure 3:**
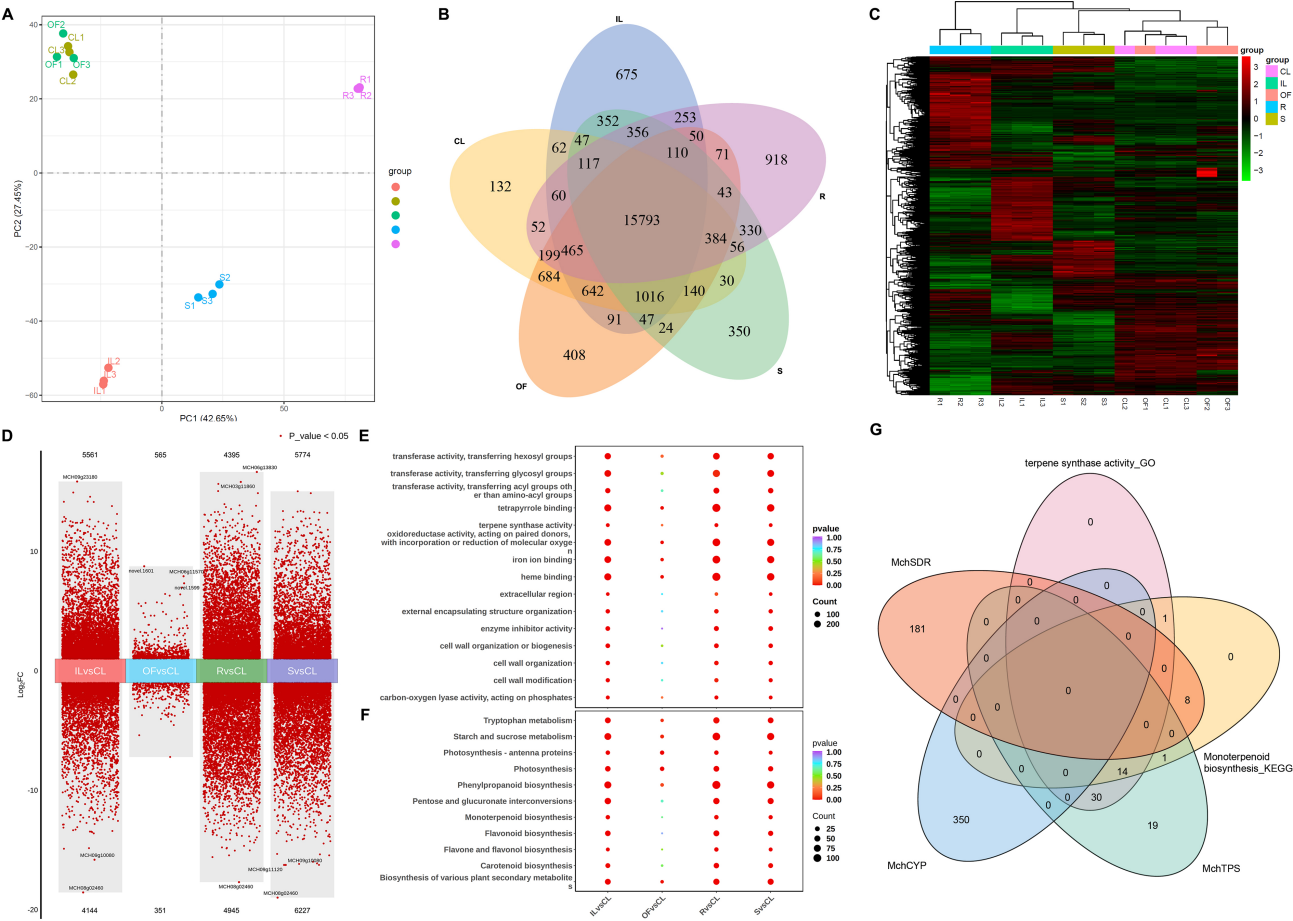
Transcriptomic analyses of OF, CL, IL, S, and R tissues. **(A)** Principal component analysis (PCA) of the RNAseq data. OF, CL, IL, S, and R represented full-bloom flowers, flower buds, leaves, stems and roots, respectively. **(B)** Venn map of co-expression genes (FPKM>1) in different tissues. **(C)** Heat map of the clustered DEGs. **(D)** Volcano map of DEGs in different compare groups. GO **(E)** and KEGG **(F)** enrichment results of DEGs. **(G)** Venn map of terpene synthase activity term from GO, monoterpenoid biosynthesis term from KEGG, and potential candidate genes of *MchTPS*, *MchCYP* and *MchSDR* from the genome. Subfigures **(A–C)** and **(G)** were drawn by TBtools, and subfigures **(D, E)** were formed using the Metware Cloud, a free online platform for data analysis (https://cloud.metware.cn).

Principal Component Analysis (PCA) was employed to estimate the differences between groups and the replicates within groups. Groups of IL, S and R were clustered and separated obviously from each other in the principal component space (**Figure 3A**), indicating that they were significantly different. However, groups of OF and CL were concentrated together but distant from the above groups, indicating that the inflorescence tissues no matter OF or CL shared similar transcription pattern. A total of 15, 793 genes were coexpressed in these five groups (**Figure 3B**). Compared with CL, ~5, 243 genes were up-regulated and ~5, 105 were down-regulated in IL, S and R groups averagely, except for OF which contained only 916 DEGs (**Figures 3C, D**). GO enrichment analysis showed that DEGs were significantly enriched and annotated into 115 GO terms including 35 in biological processes (BP), 18 in cell recognition (CC) and 62 in molecular functions (MF) in all compare groups. A total of 17 GO terms were significantly enriched in both SvsCL and ILvsCL groups. Furthermore, terpene synthase activity (GO:0010333) was significantly enriched in ILvsCL, SvsCL and RvsCL groups, indicating terpenoid biosynthesis was diversified in different tissues (**Figure 3E**). KEGG analysis of the DEGs revealed 40 biological processes were enriched for different tissues. Among them, 11 terms were simultaneously enriched in all ILvsCL, SvsCL and RvsCL compare groups, such as “Tryptophan metabolism”, “Phenylpropanoid biosynthesis”, “Monoterpenoid biosynthesis”, “Flavonoid biosynthesis” and “Carotenoid biosynthesis” (**Figure 3F**), implying that the DEGs were mainly associated with plant secondary metabolites biosynthesis.

### 3.3 Identification of γ-terpinene synthase in *M. chinensis*

**Figure 4:**
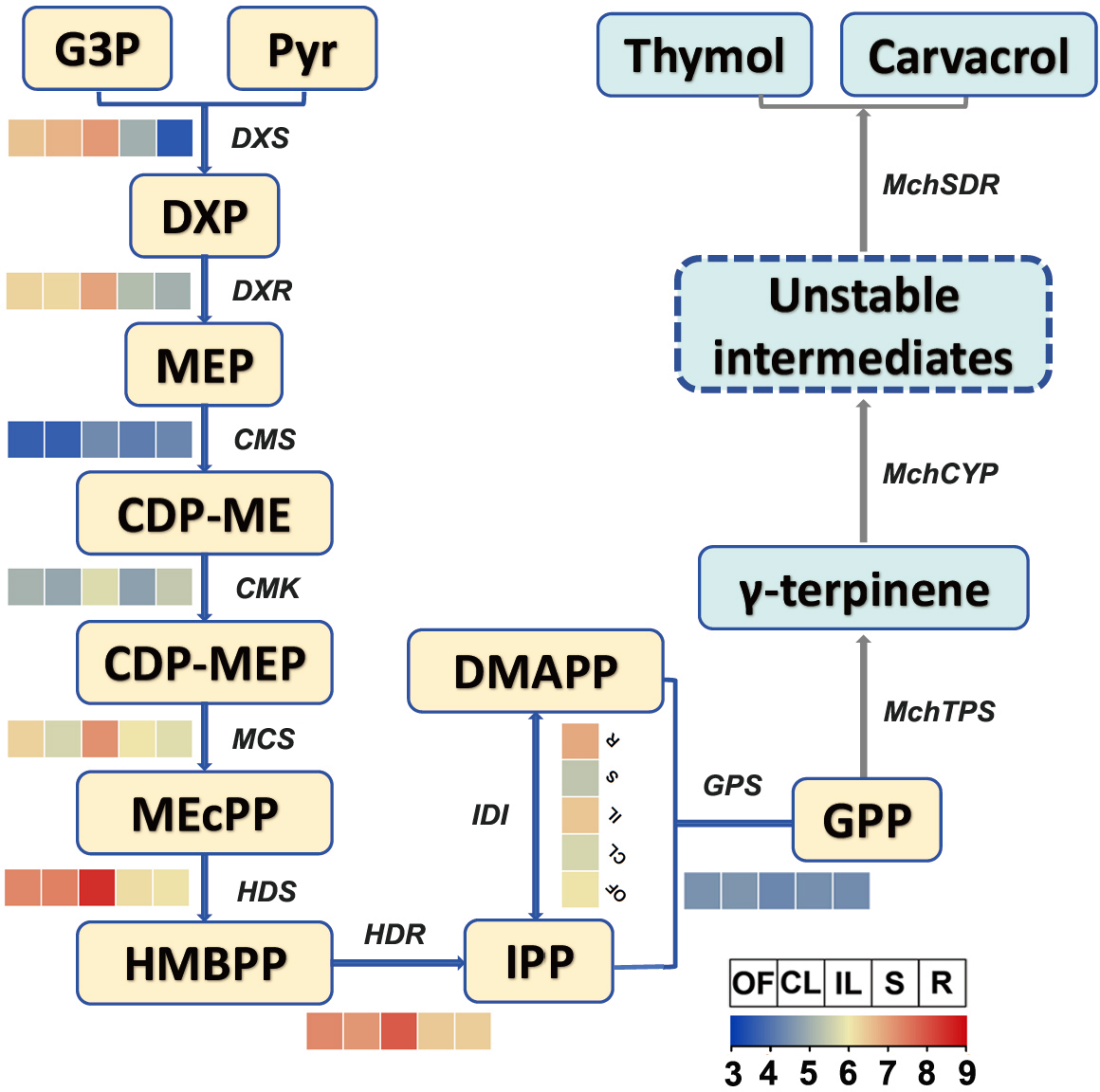
Diagram of thymol and carvacrol biosynthesis pathway in *M. chinensis*. The classical upstream of monoterpenoids biosynthesis pathway was highlighted in yellow. The transcription levels of the enzyme genes were calculated using FPKM, and the average value of three biological replicates of each tissue was shown as a heatmap. The specific downstream of thymol and carvacrol biosynthesis pathway was highlighted in blue.

The typical upstream pathway of monoterpenoids includes the enzymes of DXS (1-Deoxyxylulose 5-Phosphate Synthase), DXR (1-Deoxy-D-Xylulose 5-Phosphate Reductoisomerase), CMS (4-Diphosphocytidyl-2-C-Methyl-D-Erythritol Synthase), CMK (4-Diphosphocytidyl-2-C-Methyl-D-Erythritol Kinase), MCS (2-C-Methyl-D-Erythritol 2,4-Cyclodiphosphate Synthase), HDS (4-Hydroxy-3-Methylbut-2-Enyl Diphosphate Synthase) and HDR (4-Hydroxy-3-Methylbut-2-Enyl Diphosphate Reductase) on MEP pathway (Lichtenthaler, 1999), IDI (Isopentenyl Diphosphate Isomerase) and GPS, which flows to the formation of geranyl pyrophosphate (GPP) (**Supplementary Table 3**). Among them, the expressions of DXS, DXR, HDS and HDR showed similar patterns with the distributions of thymol and carvacrol in those tissues (**Figure 4**), which were abundant in flowers and leaves while scarce in stems and roots.

The downstream of thymol and carvacrol synthesis has not been elucidated in this alpine *M. chinensis*. The most likely first step is the formation of γ-terpinene from GPP (Lichtenthaler, 1999; Newman and Chappell, 1999), the general precursor of monoterpenoids. The enzymes catalyzing this reaction belong to the *TPS* family. To identify the *MchTPSs* (*TPS* in *M. chinensis*), the HMM search was conducted using the conserved domain of *TPS* family (PF01397, PF03936) and local BLASTP against the genome was performed using *TvTPS2*, *OvTPS2* and *TcTPS2* as queries. Finally, 65 *MchTPSs* were retrieved, and their protein lengths ranged from 94 to 818 amino acids (aa), with molecular weights from 10.98 to 92.92 kDa, pI values from 4.70 to 9.16, and predicted grand average of hydropathicity (GRAVY) values from -0.676 to -0.105, indicating that all *MchTPSs* were hydrophilic (**Supplementary Table 4**). Among them, 45 MchTPS genes were annotated to the GO term related to terpenoid biosynthesis and the KEGG pathway of monoterpenoid biosynthesis (**Figure 3G**).

**Figure 5:**
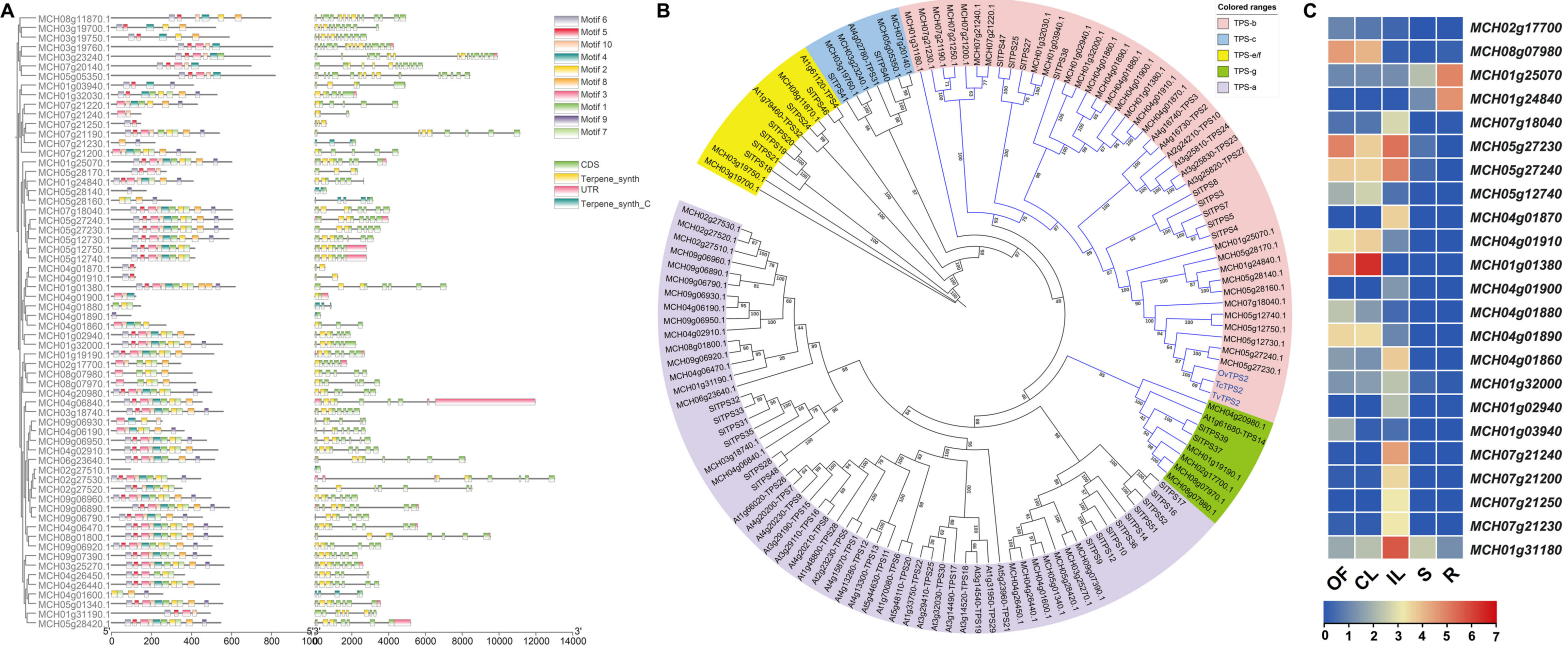
Analysis of *MchTPSs* candidate genes. **(A)** The conserved domains and gene structures of *MchTPSs*. **(B)** ML phylogenetic tree of *MchTPSs*. MchTPSs were compared with *TPSs* in *A. thaliana* (At) and *S. lycopersicum* (Sl) as well as three γ-terpinene synthase (*OvTPS2*, *TvTPS2*, *TcTPS2*, marked in blue). The bootstrap was set as 1000 and the tree was drawn to scale. **(C)** Expression levels of *MchTPSs* in different tissues. OF, full-bloom flowers; CL, flower buds; IL, leaves; S, stems; R, roots.

To investigate the evolutionary relationship and predict their functions, MchTPSs were first aligned with the TPSs from *A. thaliana* (At) and *S. lycopersicum* (Sl), and then an Maximum Likelihood (ML) phylogenetic tree was constructed. MchTPSs were categorized into five clades including TPS-a, TPS-b, TPS-c, TPS-e/f and TPS-g. A total of 33 MchTPSs was clustered into TPS-b and TPS-g clades, in which members are mainly monoterpene synthases. Excluding the incomplete and non-expressed ones, the expression patterns of the rest 23 *MchTPSs* were analyzed. The expressions of 14 genes including *MCH05g27230* were high in flowers and leaves and were almost undetectable in stems and roots, which was consistent with the tissue-specific distribution patterns of thymol and carvacrol. Furthermore, *MCH05g27230* was predicted to localized in the plastid (**Supplementary Table 4**) and showed the closest phylogenetic relationship with the γ-terpinene synthase OvTPS2 (**Figure 5**), indicating that *MCH05g27230* may play a similar role in catalyzing the synthesis of γ-terpinene in *M. chinensis*. In addtion, *MCH05g27240* which shared the same CDS sequence and cluster together with *MCH05g27230*, maybe a paralog of *MCH05g27230* from tandem duplication. Thus *MCH05g27230* was used as the representative one in following enzyme function elucidation.

### 3.4 Identifications of *MchCYPs* and *MchSDR*

The clues from *T. vulgaris* and *O. vulgare* hint that *CYP* and *SDR* gene families in *M. chinensis* participate in the further conversion of γ-terpinene to thymol and carvacrol sequentially. Additionally, *CYP* and *SDR* have been reported to be involved in the modifications of multiple terpenoids such as menthol and artemisinin (Croteau et al., 2005; Judd et al., 2019). To mine the *MchCYP* candidates from the *M. chinensis* genome, the conserved domain of CYP family (PF00067) and CYP71D179 from *T. vulgaris* were used as queries. And for *MchSDR* genes, PF00106, PF01370, PF01073 and TvSDR1 were used.

**Figure 6:**
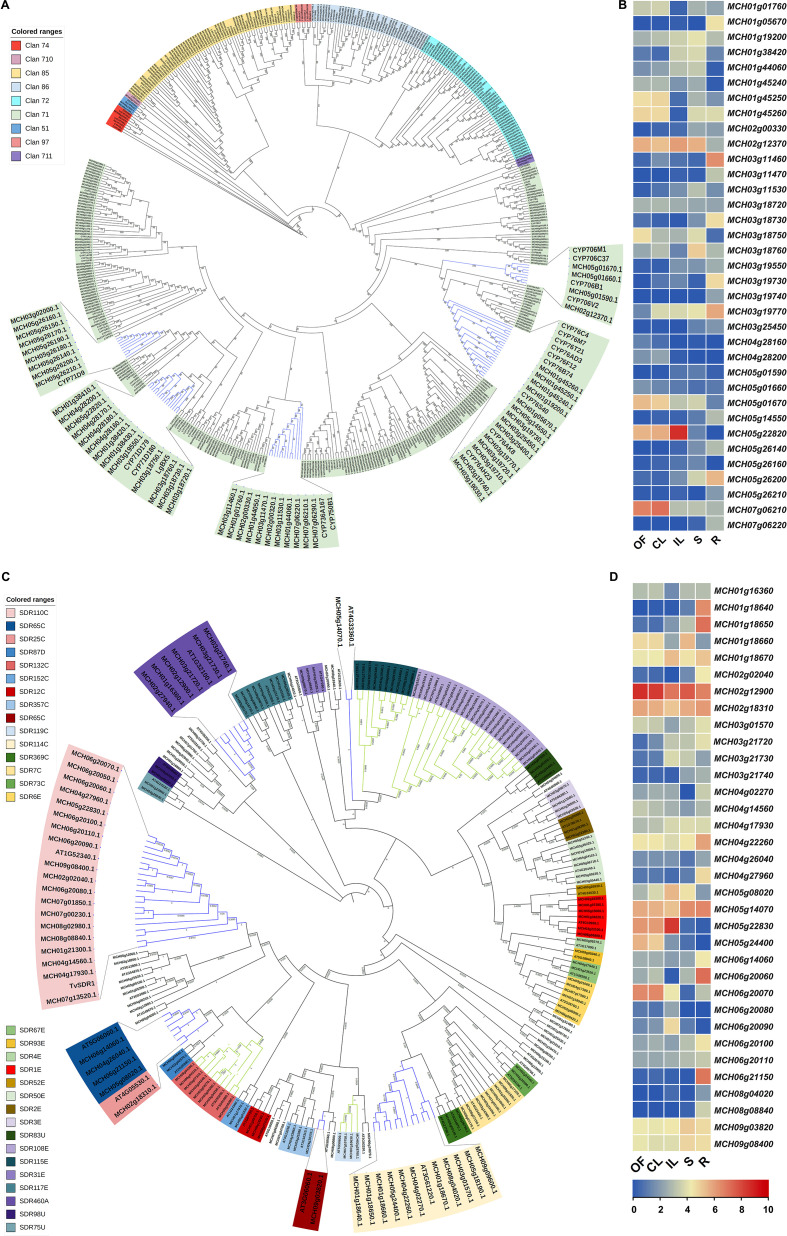
Analysis of *MchCYP* and *MchSDR* candidate genes. **(A)** Phylogenetic tree of MchCYPs. Blue branches associated with terpenoid biosynthesis. ML phylogenetic tree was constructed by comparing with the representative CYP peptides from other species with 1000 bootstrap replicates. The tree was drawn to scale. **(B)** Expression profiles of *MchCYP* genes across different tissues. **(C)** Phylogenetic tree of MchSDRs. Blue branches associated with terpenoid biosynthesis; green branches linked to secondary metabolism. Neighbor-Joining phylogenetic tree was constructed by comparing with the representative SDR genes from other species with 100 bootstrap. The tree was drawn to scale. **(D)** Expression profiles of *MchSDRs* across different tissues. OF, full-bloom flowers; CL, flower buds; IL, leaves; S, stems; R, roots.

A total of 350 *MchCYPs* were identified. The lengths of MchCYPs ranged from 64 to 756 amino acids (aa), their molecular weights from 7.5 to 85.27 kDa, pI values from 4.75 to 10.24, and the predicted grand average of hydropathicity (GRAVY) values from -0.72 to 0.495 (**Supplementary Table 5**). This indicates that most of MchCYPs were hydrophilic except that 28 out of 350 were hydrophobic. They were then aligned with the representative CYP genes of each CYP family downloaded from the Plant P450 Database (erda.dk/public/vgrid/PlantP450/). Previous studies have demonstrated that the oxidation of terpenoids is primarily catalyzed by the CYP71 clade, specifically members of the CYP76, CYP706, CYP736, CYP750 families. 50 MchCYPs belong to those families. Excluding the incomplete and low-expressed genes, the expression patterns of the remaining 35 MchCYPs were shown. Only 18 *MchCYPs* including *MCH05g2280* were highly expressed in flowers and leaves while lowly expressed in stems and roots. Among these, *MCH03g18750* exhibited a close phylogenetic proximity and similar subcellular localization pattern in endoplasmic reticulum to the previously characterized TvCYP71D179 (**Figures 6A, B**; **Supplementary Table 5**), suggesting its potential involvement in thymol and carvacrol biosynthesis in *M. chinensis*.

A total of 189 *MchSDRs* were identified from the genome of *M. chinensis*. Their lengths varied from 94 to 1008 aa, with molecular weights from 10.08 to 114.31 kDa, pI values from 4.88 to 9.95, and the predicted grand average of hydropathicity (GRAVY) values from -0.666 to 0.55, indicating that the hydrophilic properties of MchSDRs are very diverse (**Supplementary Table 6**). Among them, 8 MchSDRs were annotated to the KEGG pathway of monoterpenoid biosynthesis (**Figure 3G**). Previous studies have shown that SDR25C, SDR110C, SDR114C, SDR460A families, and the AT4G33360.1 clade are primarily involved in the dehydrogenation/reduction reactions of terpenoid compounds. Although most reported members of the SDR65C family participate in tropinone alkaloid biosynthesis, some genes within this family are also implicated in terpenoid metabolism. Among these, 43 MchSDRs were clustered into the aforementioned families (**Figure 6C**; blue branches). After excluding the genes that were low-expressed genes, structurally incomplete or predominantly expressed in roots, 23 *MchSDRs* were selected for further investigation (**Figures 6C, D**). MCH04g17930 and MCH04g14560 may participate in the biosynthesis of thymol and carvacrol in *M. chinensis* because of their close phylogenetic relationships with the reported TvSDR1. However, MCH04g17930, MCH04g14560 and TvSDR1 exhibited distinct subcellular localization patterns in Nucleus, Mitochondrion and Cytoplasm, respectively (**Supplementary Table 6**).

### 3.5 Functional verification and BGCs analysis of *MchTPSs* and improvement of γ-terpinene titer

**Figure 7:**
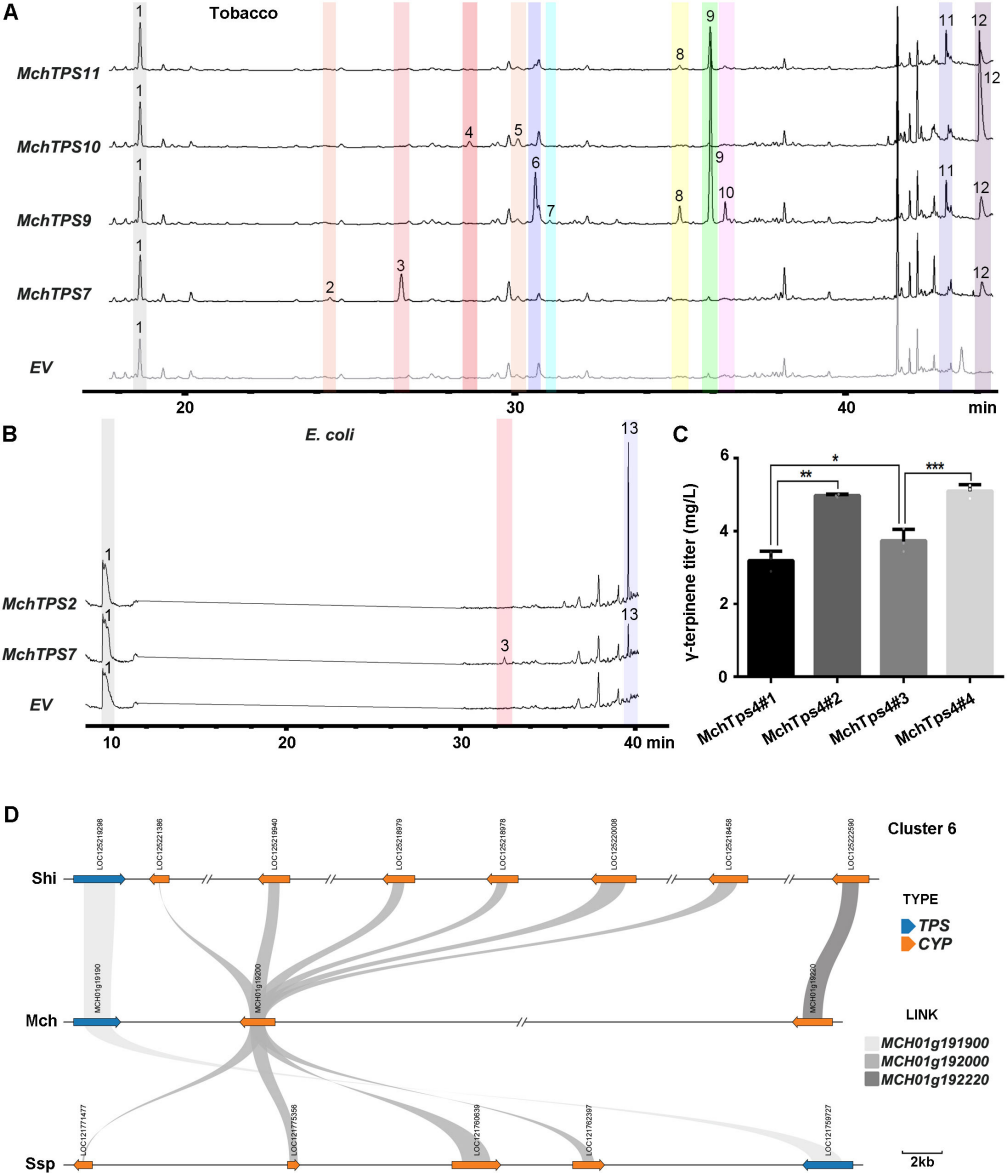
Functional characterization and BGCs analysis of *MchTPSs*. **(A)** Functional characterization of *MchTPSs* in *N. benthamiana*. **(B)** Functional verification of *MchTPSs* in *E.coli*. **(C)** Improvement of γ-terpinene yield in *E.coli* chassis. MchTPS4#1, the original sequence of MchTPS4; MchTPS4#2, the original sequence without signal peptide; MchTPS4#3, optimized sequence according to *E.coli* codon; MchTPS4#4, optimized sequence without signal peptide. **(D)** Collinearity analysis of cluster 6 compared with *Salvia hispanica* (Shi) and *Salvia splendens* (Ssp) genomes. Lines indicate orthologous genes. The chromosomes were drawn to the scale. Compounds list: 1, Nonyl acetate; 2, β-caryophyllene; 3, α-caryophyllene; 4, α-muurolene; 5, isolongifolene; 6, γ-cadinene; 7, γ-muurolene; 8, β-cadinene; 9, (+)-epi-bicyclosesquiphellandrene; 10, α-cadinol; 11, geranyl-α-terpinene; 12, aromandendrene; 13, γ-elemene.

The functions of *MchTPS* candidate genes were verified in *E. coli* or tobacco system. The major products of *MchTPS7* were γ-elemene (1.12 mg/L) and α-caryophyllene (1.21 mg/L) in *E. coli* and two more sesquiterpenoids of aromandendrene (9.21 μg/g FW, FW represents fresh weight of plant sample) and β-caryophyllene (2.94 μg/g FW) in tobacco (**Figures 7A, B**). *MchTPS9* yielded multiple products including γ-cadinene (52.96 μg/g FW), (+)-epi-bicyclosesquiphellandrene (96.06 μg/g FW), geranyl-α-terpinene (27.73 μg/g FW), α-cadinol (15.28 μg/g FW) and aromandendrene (10.25 μg/g FW) (**Figure 7A**). The main product of *MchTPS10* was aromandendrene with the yield of 57.64 μg/g FW (**Figure 7A**). The products of *MchTPS11* were mainly (+)-epi-bicyclosesquiphellandrene (18.02 μg/g FW) and geranyl-α-terpinene (18.69 μg/g FW) (**Figure 7A**; **Supplementary Table 4**). MchTPS2 was a sesquiterpene synthase and mainly produced γ-elemene (5.9 mg/L) (**Figure 7B**). The function of *MchTPS4* was verified in *E. coli* and turned out to generate γ-terpinene which was the first committed precursor of carvacrol and thymol (**Supplementary Figure 3**). The initial titer of γ-terpinene was trace until the MEP pathway was strengthened by introducing *pIRS*, the titer reached 1.48 mg/L. To enhance the titer, the gene structure of *MchTPS4* and culture conditions were further optimized. Specifically, glycerol and pyruvic acid were added to the medium to provide carbon source and metabolic substrate; meanwhile, the signal peptide of *MchTPS4* was deleted and its codon was optimized. After codon optimization, the γ-terpinene content reached 3.72 mg/L. The yield increased to 5.09 mg/L by the further deletion of the signal peptide, which was 3.45-fold higher than the initial titer (**Figure 7C**).

The enzymes, catalyzing the distinct reactions in a plant specialized metabolite pathway, localized physically close to each other are nominated as Biosynthetic Gene Clusters (BGCs) (Qiao et al., 2025). BGCs provide a novel approach to clarify plant specialized metabolite pathways. We identified a total of 61 clusters related to plant specialized metabolism. Of these, clusters 1, 6, 11, 24, 25, 27 and 56 belonged to the “Terpene Type”. Additionally, the products or substrates of clusters 8, 13, 34, 39 were associated with monoterpenoids (**Supplementary Table 8**). *MchTPS2* and two *MchCYPs* (*MCH01g19200* from Clan 71; *MCH01g19220* from Clan 86) were members of sesquiterpenoid cluster 6. This cluster was syntenic between *S. divinorum* and *S. hispanica*, whereas the homologous gene of *MCH01g19220* was lost in *S. splendens*. TPS typically existed as single-copy genes across these compared genomes. However, *MCH01g19200* in *M. chinensis* corresponded to four homologous genes in the other three plant species (**Figure 7D**; **Supplementary Table 8**), implying that duplication events have occurred in these lineages. *MchTPS11* was located in cluster 56 along with three more *MchTPSs* (*MCH09g06920*; *MCH09g06950*; *MCH09g06960*) and one glycosyltransferase (*MCH09g06970*) (**Supplementary Table 8**), indicating that cluster 56 may participate in terpene synthesis and modification. The remaining functionally verified *MchTPSs* including *MchTPS4* were not clustered. Interestingly, in addition to the BGCs, we found that *MchTPS*, *MchCYP* and *MchSDR* were distributed more frequently on chromosomes 1 (**Supplementary Tables 4**, **5**), suggesting a couple of tandem duplication events may contribute to the emergence of new genes.

### 3.6 Phylogenetic and comparative genomic analysis of *M. chinensis*

**Figure 8:**
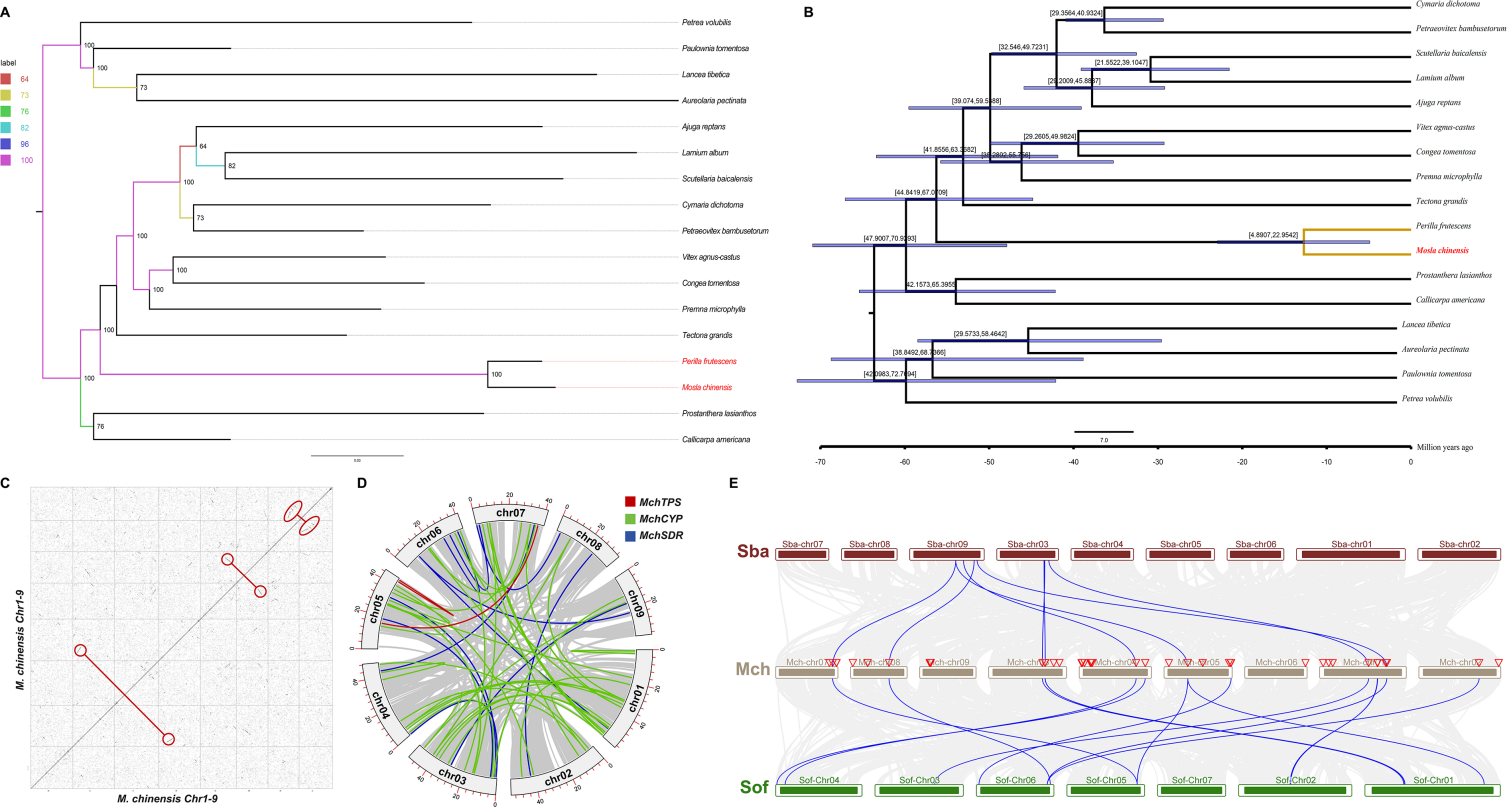
Evolutionary analysis of the *M. chinensis* genome. **(A)** Maximum likelihood phylogeny inferred from concatenated single-copy orthologous genes. **(B)** Phylogenetic time tree estimating divergence times within Lamiaceae, based on one representative species sampled per subfamily. **(C)** Dot plot of the assembled *M. chinensis* chromosomes. The dotted black lines may indicate paralogues produced by segmental duplication or WGD, such as those circled in red. **(D)** intergenomic synteny patterns of *MchTPS* (red), *MchCYP* (green) and *MchSDR* (blue) in *M. chinensis* genome. **(E)** Comparison of chromosomes between *M. chinensis*, *S. baicalensis* and *S. officinalis*. The synteny pattern of *TPS* genes were shown in blue line.

To elucidate the taxonomic position of *M. chinensis*, we chosen 12 subclades representing the core diversity of Lamiaceae, with four outgroups from Lamiales (*Aureolaria pectinata*, *Lancea tibetica*, *Petrea volubilis* and *Paulownia tomentosa*). From transcriptomes, we identified 141 single-copy orthologous nuclear genes (SCGs) for subsequent analyses. Concatenated analysis of SCGs under maximum likelihood yielded a fully resolved phylogeny (**Figure 8A**). *M. chinensis* was found to be a sister lineage to *P. frutescens*, which formed a maximally supported clade (BS = 100%). Molecular dating with MCMCTREE placed their divergence in the Miocene, the divergence of *M. chinensis* and *P. frutescens* occurred at approximately 12.69 million years ago (Mya) with a 95% confidence from the range of 4.89-22.95 Mya (**Figure 8B**).

Collinearity analyses were conducted within *M. chinensis* or with the closely related species of *S. baicalensis* and *S. officinalis*. The intragenomic collinear analysis detected at least one whole genome duplication (WGD) event in *M. chinensis* genome (**Figure 8C**). And for the *MchTPSs* family, collinearity may form from tandem duplication in chromosome 05 (pair of *MchTPS4* and *MCH05g05350*) or from WGD or segmental duplication between chromosome 05 and chromosome 07 (pair of *MCH05g28160* and *MCH07g20140*) (**Figure 8D**). The intergenomic collinear analyses identified that two paralogous segments in *M. chinensis* corresponded to one orthologous region in *S. baicalensis* but that was one-to-one between *M. chinensis* and *S. officinalis*. These results suggested that *M. chinensis* probably had a species-specific WGD event after it split away from the common ancestor with *S. baicalensis* (**Figure 8E**). Furthermore, the aforementioned *MchTPS* pairs within the *M. chinensis* were also syntenic between *M. chinensis* and *S. baicalensis*; and the whole *MchTPS* pairs between *M. chinensis* and *S. baicalensis* were included in the syntenic gene pairs between *M. chinensis* and *S. officinalis* too (**Supplementary Table 7**). These results supported that *S. officinalis* was the earliest diverging group among these three species during the evolutionary process and the phylogenetic relationship between *M. chinensis* and *S. baicalensis* was closer. These syntenic *MchTPS* genes have been stably retained during evolution indicating their core physiological functions in plant.

## 4 Discussion

*M. chinensis* is a traditional Chinese medicinal plant primarily distributed in southern China. However, its evolutionary and genetic studies have been hindered due to the lack of high-quality genome information. In this study, we present a high-quality chromosome-level genome assembly of *M. chinensis* from the alpine ecotype, using PacBio (long reads) and Illumina HiSeq (short reads) sequencing technologies, combined with Hi-C scaffolding. The final assembled genome size was 444.26 Mb (2n=18), slightly smaller than the estimated sizes of 465.74 Mb (survey) and 481.90 Mb (flow cytometry). A particular focus of our study was on the mining of terpenoid biosynthesis enzymes, and we extensively characterized the functions of the sesquiterpene synthase genes *MchTPS2*, *MchTPS7*, *MchTPS9–11* and monoterpene synthase *MchTPS4*. Notably, *MchTPS4* is responsible for γ-terpinene formation, which is the first committed compound in the synthesis of carvacrol and thymol.

We also observed the release of a genome for a *M. chinensis* sample collected from Jiangxi (This variant is briefly referred to as “Mc” while our alpine variant as “Mch”) in October 2025 (Yu et al., 2025). The genome of Mc was also anchored to nine pseudochromosomes (2n=18) with a size of 426.1 Mb. We hypothesize that the difference in genome size between Mc and Mch arises because Mch is a high-altitude specimen from Zhejiang. The study of Mc verified the functions of five monoterpene synthases, including McTPS1, which is involved in γ-terpinene synthesis. A comparison of these McTPS sequences with our MchTPS sequences via BlastP revealed that McTPS1 and McTPS5 were identical to MchTPS4 and MCH01g25070, respectively. However, no highly similar matches were found for the remaining three McTPS sequences. These findings, including the variations in genome size and differences in TPS sequences, underscore the significant genomic divergence that can exist among populations of the same species from different geographic regions. Such variations likely arise from long-term local adaptation, thereby contributing to the formation of geo-authenticity in traditional Chinese medicines.

Enzyme genes involved in plant secondary metabolic pathways are often organized into “operon-like” clusters within genomes, offering new opportunities for pathway elucidation through genome mining. We conducted a thorough investigation into the BGCs of *M. chinensis* and focused on those associated with terpenoid synthesis. Previous studies have shown that the biosynthesis of carvacrol and thymol, major components in the volatile compounds of *M. chinensis*, is catalyzed sequentially by TPS, CYP, and SDR family genes (Krause et al., 2021). However, we found that none of the BGCs in *M. chinensis* contained all three enzyme types simultaneously. Additionally, due to incomplete or absent genome data for *T. vulgaris* and *O. vulgare*, in which the synthetic pathways for thymol and carvacrol have been studied, we were unable to determine whether the reported enzymes were clustered in these species. Plants produce around 10^5^ to 10^6^ diverse metabolites. In contrast to BGCs, most synthetic genes of plant metabolites are dispersed (non-BGCs) in the genome (Polturak and Osbourn, 2021). BGCs of plant secondary metabolites are highly diverse and rapidly evolving in the genome. Even though, little is known about how BGCs originate, more evidence imply that the formation and maintenance of BGCs require specific and consistent particular selective pressures to improve plant environmental fitness (Liu et al., 2020; Osbourn, 2010). Therefore, the plausible explanations for the scattered distribution of TPS, CYP, and SDR in *M. chinensis* genome are that these genes have existed for a long time but lacked sufficient selective pressure to form a TPS-CYP-SDR cluster; or that these genes are newly emerged and too evolutionarily young to assemble into clusters; or that they once formed a cluster in the past but the cluster collapsed due to shifts in selective pressures. In most cases, TPS was found to cluster with CYP, and in one BGC, TPS was clustered with SDR (**Supplementary Table 8**). This suggests that TPS and CYP likely co-evolved in the evolutionary trajectory, with the incorporation of SDR potentially contributing to the diversity of terpenoids.

This phenomenon may provide a plausible explanation for why thymol and carvacrol co-occur in the genera *Mosla*, *Origanum*, and *Thymus* within the Nepetoideae subfamily (Lee et al., 2023; Xin et al., 2023). Notably, within the *Mosla* genus, they appear simultaneously only in *M. chinensis* and its variety *M. chinensis* ‘Jiangxiangru’ (**Supplementary Figure 2**). Beyond the Lamiaceae family, the occurrence of thymol and carvacrol is restricted to only a few genera, such as *Trachyspermum* in Apiaceae and *Lippia* in Verbenaceae (Krause et al., 2021). These insights open intriguing possibilities for unraveling the evolutionary mechanisms that underlie the species- and genus-specific distribution of these phenolic monoterpenes, especially with the increasing availability of genomic data.

Our phylogenomic analysis based on 141 single-copy nuclear genes positioned *M. chinensis* as a sister lineage to *Perilla frutescens*, with their divergence estimated in the Miocene (~12.69 Mya). This result provides strong molecular evidence supporting the close evolutionary relationship between these two genera within Lamiaceae, consistent with the plastid phylogeny framework proposed by Zhao et al., who achieved robust resolution at both deep and shallow nodes using 79 plastid genes across 12 subfamilies (Zhao et al., 2021). The congruence between nuclear and plastid phylogenies strengthens confidence in the inferred topology and suggests that *M. chinensis* and *P. frutescens* share a relatively recent common ancestor within the Nepetoideae clade. Moreover, our data provide additional evidence of a more recent divergence, likely influenced by Miocene climatic fluctuations and niche differentiation, which could have promoted adaptive diversification in these aromatic species.

Comparative collinearity and duplication analyses revealed at least one species-specific whole-genome duplication (WGD) event in *M. chinensis* after its split from *S. baicalensis*, along with evidence of tandem and segmental duplications in the *MchTPS* gene family. These results echo the findings of Godden et al (Godden et al., 2019), who demonstrated extensive but uneven WGD occurrences (7–18 events) across Lamiaceae, particularly within Nepetoideae, suggesting that polyploidy has been a recurrent and lineage-specific driver of diversification. Furthermore, the expansion of TPS genes in *M. chinensis* aligns with the chemical-genomic insights of Boachon et al (Boachon et al., 2018), who linked terpene chemodiversity primarily to gene family expansion rather than enzyme promiscuity. Together, these comparisons indicate that the combination of lineage-specific genome duplication and functional retention of *MchTPS* genes has likely contributed to the unique metabolic diversity of *M. chinensis*, reinforcing the role of gene duplication as a key evolutionary force shaping specialized metabolism in Lamiaceae.

## 5 Conclusion

The chromosome-scale genome of alpine *M. chinensis*, assembled using SMRT sequencing and Hi-C technologies, serves as a valuable genomic resource for precision breeding of this high-value medicinal plant. The 444.26 Mb genome, characterized by high accuracy and completeness, reveals genomic features including high repetitive sequence content and whole-genome duplication, which may contribute to the plant’s resilience in extreme alpine environments. Multi-tissue metabolomic and transcriptomic analyses identified key biosynthetic genes for terpenoids, with *MchTPS4* playing a central role in γ-terpinene synthesis. Metabolic engineering further demonstrated the potential for enhancing γ-terpinene production. Additionally, this study highlights the chromosomal clustering of key metabolic genes and provides insights into the evolutionary processes driving the biosynthesis of specialized metabolites. Comparative genomics analyses uncovered a whole-genome duplication event that shaped the retention of metabolic genes, thereby offering deeper insights into metabolic evolution in the Lamiaceae family. Collectively, this study lays the foundation for future molecular breeding efforts and advances our understanding of terpenoid biosynthesis in extremophytes.

## Data Availability

The assembled genome data could be accessed through SAMC7447979. The transcriptome data are available under accession numbers SAMC7443207toSAMC7443221, which have been uploaded to the China National Center for Bioinformation (CNCB, https://ngdc.cncb.ac.cn). The annotations are available at figshare (https://doi.org/10.6084/m9.figshare.31979658). The raw reads or bam files generated here have been submitted to the NCBI Bio Project database (https://www.ncbi.nlm.nih.gov/bioproject/) under accession number PRJNA1355007. The data and materials used and/or analyzed in this study are available from the corresponding author on reasonable request.

## Supplementary material

Supplementary Figure 1 Genome size estimation and karyotype analysis of *M. chinensis*.

Supplementary Figure 2 Heatmap of the distribution of volatiles from the leaves of different plants. *Elsholtzia zhongyangii* from Genus *Elsholtzia*; *M. dianthera*, *M. scabra*, *M. soochouensis*, *M. cavaleriei* and *M. chinensis* from Genus Mosla.

Supplementary Figure 3 TIC and mass spectrometry of standard substance and products of *MchTPS4*.

Supplementary Table 1 Secondary calibration nodes and constraints used in MCMCTREE analysis.

Supplementary Table 2 Transcriptome data statistics.

Supplementary Table 3 Upstream genes of terpenoids biosynthesis in *M. chinensis*.

Supplementary Table 4 MchTPS protein parameters and the yield of products in *E. coli* or tobacco.

Supplementary Table 5 MchCYP protein parameters.

Supplementary Table 6 MchSDR protein parameters.

Supplementary Table 7 Data for synteny analysis intra and inter genomes.

Supplementary Table 8 BGCs information.
