# Supplementary figures and images for: Chromosome-level genome of Mosla chinensis from alpine ecotype provides insights into terpenoid biosynthesis and germplasm exploration
Source: Front Plant Sci. 2026 Jun 16;17:1819008. doi: 10.3389/fpls.2026.1819008 (PMC13315002; doi:10.3389/fpls.2026.1819008)

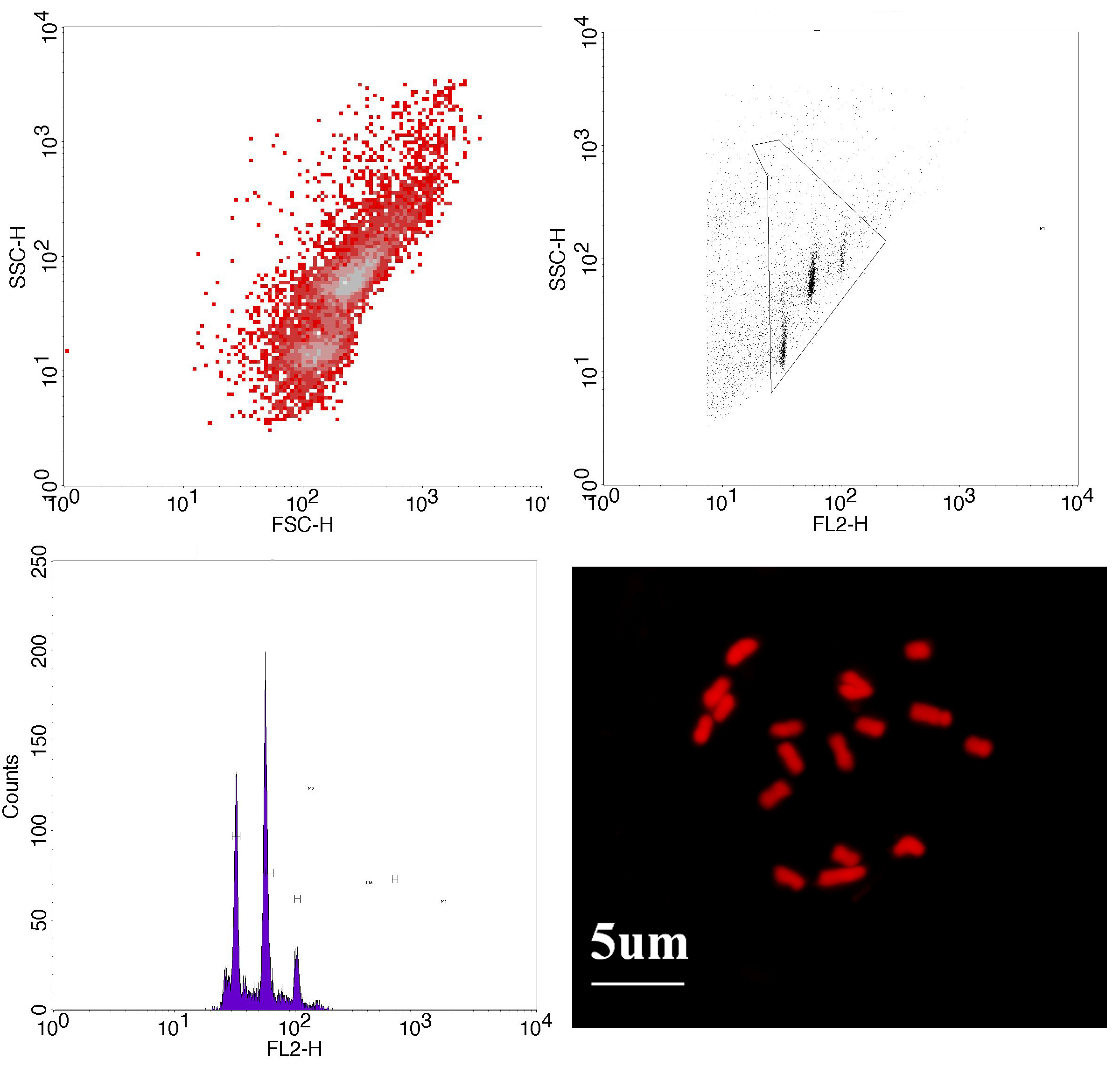

Supplement: Supplementary Figure 1 — Genome size estimation and karyotype analysis of M. chinensis. [file Image1.jpeg]

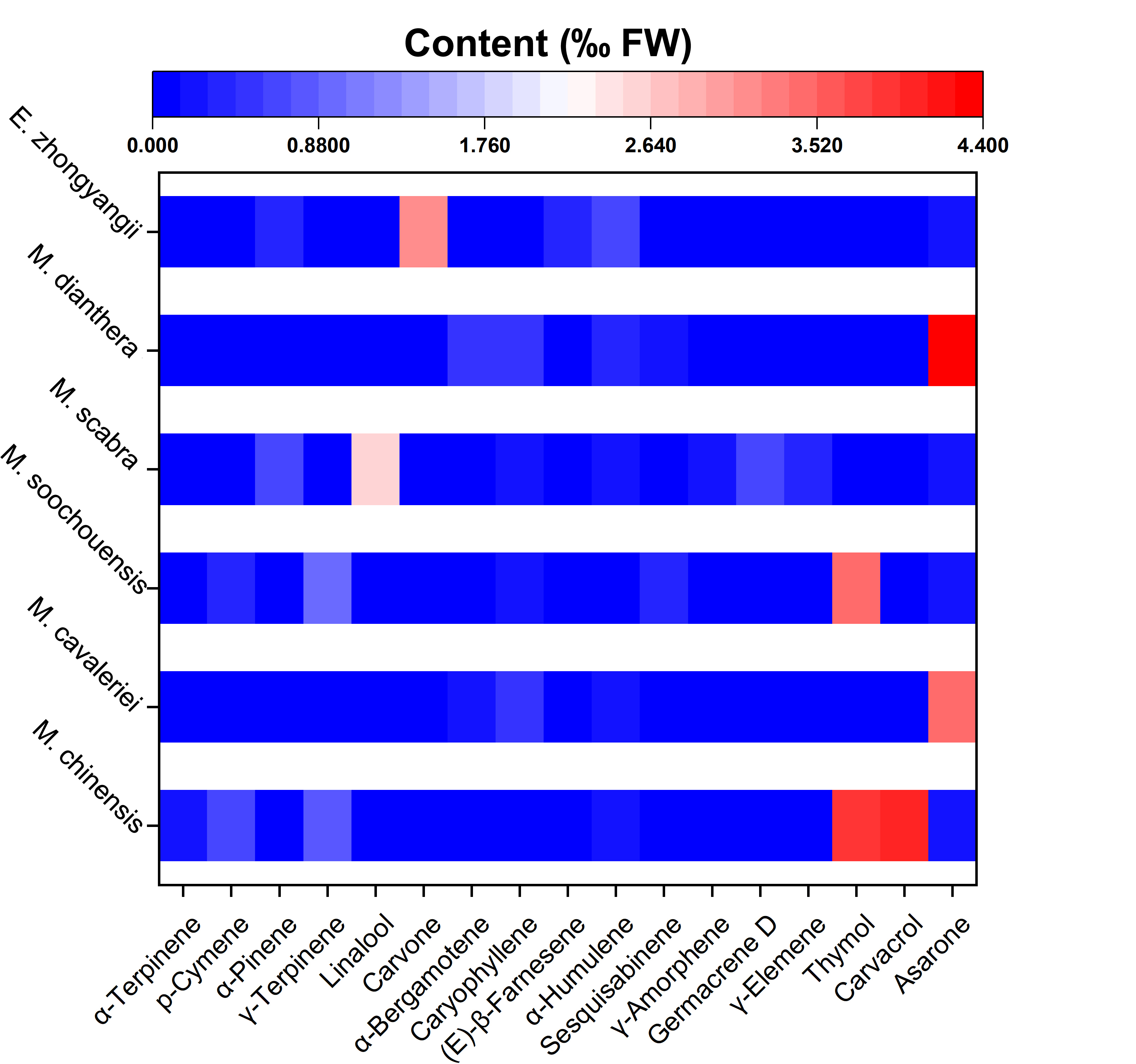

Supplement: Supplementary Figure 2 — Heatmap of the distribution of volatiles from the leaves of different plants. Elsholtzia zhongyangii from Genus Elsholtzia; M. dianthera, M. scabra, M. soochouensis, M. cavaleriei and M. chinensis from Genus Mosla. [file Image2.jpeg]

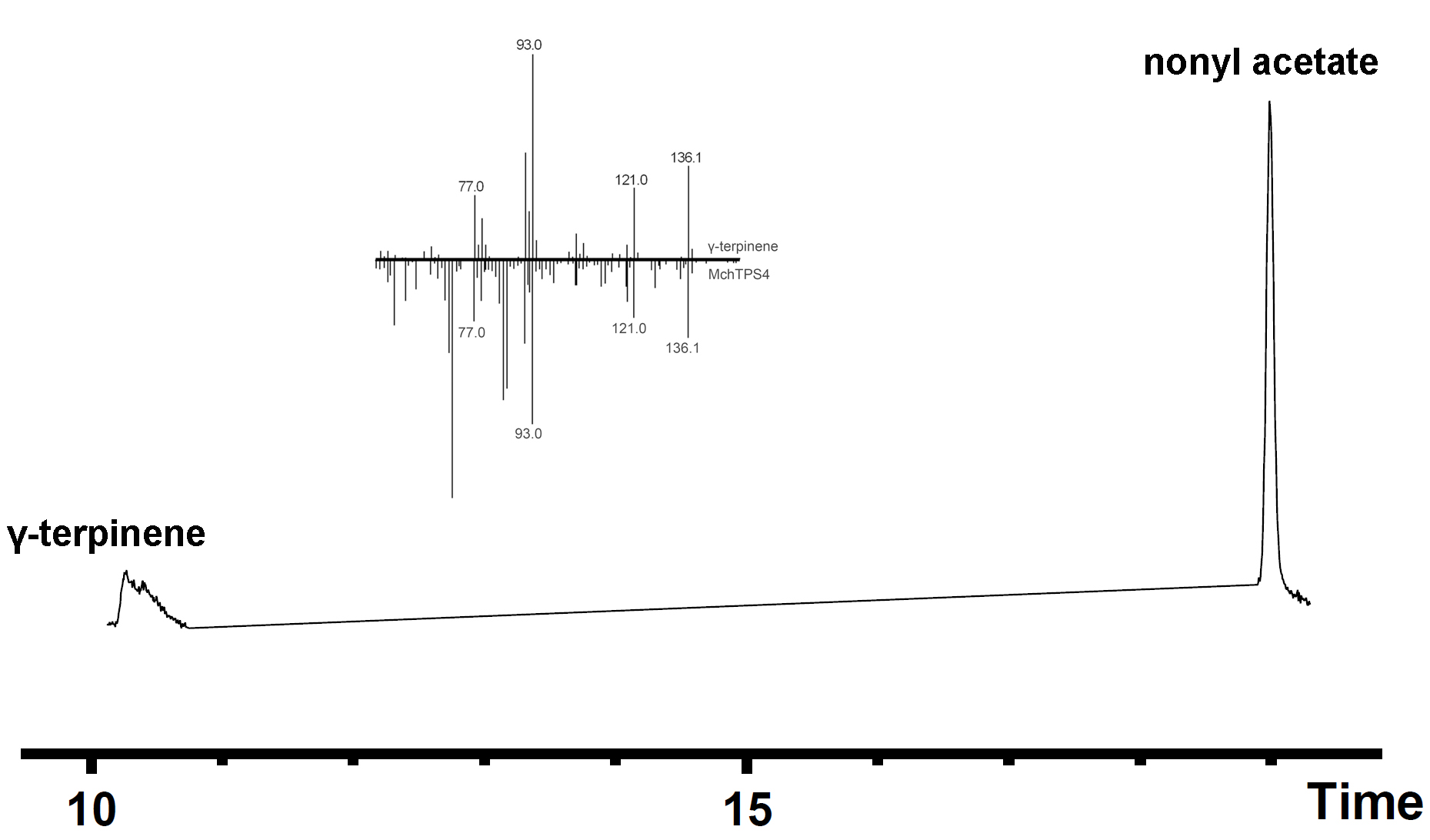

Supplement: Supplementary Figure 3 — TIC and mass spectrometry of standard substance and products of MchTPS4. [file Image3.jpeg]
