# Supplementary material for: Chromosome-level genome of Mosla chinensis from alpine ecotype provides insights into terpenoid biosynthesis and germplasm exploration
Source: Front Plant Sci. 2026 Jun 16;17:1819008. doi: 10.3389/fpls.2026.1819008 (PMC13315002; doi:10.3389/fpls.2026.1819008)
Supplement: Supplementary Table 1 — Secondary calibration nodes and constraints used in MCMCTREE analysis. [file Table1.docx]

**Supplementary table 1. Secondary calibration nodes and constraints used in MCMCTREE analysis**

| **Taxa1** | **Taxa2** | **Min age (Ma)** | **Min Age Supporting References** | **Max age (Ma)** | **Max Age Supporting References** |
| --- | --- | --- | --- | --- | --- |
| *Lancea tibetica* | *Aureolaria pectinata* | 0 | (Davies, et al. 2013) | 68.6 | (Zhang, et al. 2020) |
| *Lamium album* | *Scutellaria baicalensis* | 19 | (Li, et al. 2019) | 48.3 | (Zhang, et al. 2022) |
| *Cymaria dichotoma* | *Petraeovitex bambusetorum* | 27.2 | (Roy and Lindqvist 2015) | 40.4 | (Ramírez-Barahona, et al. 2020) |
| *Vitex agnus-castus* | *Congea tomentosa* | 13.4 | (Roy and Lindqvist 2015) | 54.9 | (Drew and Sytsma 2012) |
| *Prostanthera lasianthos* | *Callicarpa americana* | 22.4 | (Roy and Lindqvist 2015) | 66.9 | (Drew and Sytsma 2012) |

Davies, T Jonathan, et al.

2013 Phylogenetic conservatism in plant phenology. Journal of ecology 101(6):1520-1530.

Drew, Bryan T., and Kenneth J. Sytsma

2012 Phylogenetics, biogeography, and staminal evolution in the tribe Mentheae (Lamiaceae). American Journal of Botany 99(5):933-953.

Li, Hong-Tao, et al.

2019 Origin of angiosperms and the puzzle of the Jurassic gap. Nature Plants 5(5):461-470.

Ramírez-Barahona, Santiago, Hervé Sauquet, and Susana Magallón

2020 The delayed and geographically heterogeneous diversification of flowering plant families. Nature Ecology & Evolution 4(9):1232-1238.

Roy, Tilottama, and Charlotte Lindqvist

2015 New insights into evolutionary relationships within the subfamily Lamioideae (Lamiaceae) based on pentatricopeptide repeat (PPR) nuclear DNA sequences. American Journal of Botany 102(10):1721-1735.

Zhang, Caifei, et al.

2020 Asterid Phylogenomics/Phylotranscriptomics Uncover Morphological Evolutionary Histories and Support Phylogenetic Placement for Numerous Whole-Genome Duplications. Molecular Biology and Evolution 37(11):3188-3210.

Zhang, Qiang, et al.

2022 New insights into the formation of biodiversity hotspots of the Kenyan flora. Diversity and Distributions 28(12):2696-2711.
